# Sternal Dislocation and Associated Lung Lobe Hernia in a Cat

**DOI:** 10.1155/2024/3719641

**Published:** 2024-05-14

**Authors:** Martin Hamon, Philippe Haudiquet, Aurelie Bruwier

**Affiliations:** ^1^Veterinary Health Center, Kansas State University, 1800 Denison Ave, Manhattan, Kansas 66506, USA; ^2^Clinique veterinaire VetRef, 7 Rue James Watt, 49070 Beaucouze, France

## Abstract

Sternal luxation/dislocation is a rare condition and is most often the result of trauma. Medical and surgical management have been described, with scarce information regarding the best treatment option for these cases. A 1-year-old domestic shorthair cat was presented for severe sternal dislocation and a left humeral fracture. Given the displacement observed at the level of the sternum and pain associated, surgical stabilization was elected. A partial lung lobe hernia was identified during the open stabilization of the sternum. Management of the hernia and sternal luxation stabilization were performed with the aid of video-assisted thoracoscopy. The cat recovered uneventfully, and no postoperative complications were reported.

## 1. Introduction

The sternum plays a major role by protecting the heart and lungs and aids in breathing motion [1]. Sternal luxation/dislocation is a rare condition and is most often the result of trauma [2]. Only 5 cases (4 cats and 1 dog) have been specifically reported in the literature [2–5]. Medical and surgical management have been described, with scarce information regarding the best treatment option for these cases.

Lung herniation is defined as a protrusion of pulmonary tissue beyond the normal boundaries of the thoracic cavity [6]. Lung hernias can be classified according to location (cervical, thoracic, or diaphragmatic) and etiology (congenital and acquired) [7]. Acquired etiologies include iatrogenic (following surgical intervention), traumatic, pathologic (resulting from neoplasia or inflammation), or spontaneous disease [7, 8]. The most frequent cause is traumatic, particularly with bite wounds penetrating through the intercostal muscles [9].

The aim of this case report is to report the surgical management of a sternal dislocation with the aid of video-assisted thoracoscopy to help minimize iatrogenic injury. A concomitant lung lobe hernia was also treated at the same time.

## 2. Case Report

A 1-year-old male domestic shorthair cat was referred for left humeral fracture after being hit by a car 3 days previously.

On physical examination, the cat exhibited dyspnea (50 breaths/min), tachycardia (226 beats/min), and pain at the level of the sternum. A large subcutaneous hematoma was present at the ventral aspect of the thorax and was associated with a small cutaneous wound. A serum biochemical analysis revealed mild hyperglycemia (glucose 170 mg/dl, ref: 74-159), and a complete blood count revealed very mild anemia (hematocrit 22%, ref: 24-55%).

The patient was initially managed with analgesia, oxygen therapy, and intravenous fluid therapy.

**Figure 1:**
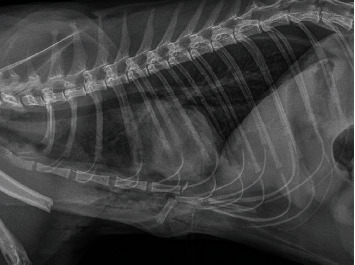
Right lateral thoracic radiograph showing severe cranioventral displacement of the 6^th^ sternebra, associated with sternal dislocation between the 5^th^ and 6^th^ sternebrae and sternal subluxation between the 6^th^ and 7^th^ sternebrae. Mild pneumothorax, mild pleural effusion, and subcutaneous emphysema are also visible. The left humeral fracture is partially visualized.

Orthogonal thoracic radiographs revealed complete sternal luxation between the 5th and 6th sternebrae with marked cranioventral displacement and angulation of the 6th sternebra. A subluxation between the 6th and 7th sternebrae, with secondary cranioventral sternal dislocation of the caudal segment, was also present (Figure 1). This resulted in cranial displacement of the ventral part of the diaphragm and a mild reduction in the volume of the thoracic cavity. Marked soft tissue swelling associated with emphysema was visible at the level of the sternum, partially superimposed over the ventral aspect of the thoracic cavity limiting the evaluation of the lungs at this level. However, patchy areas of increased pulmonary opacity in the ventral part of the lung parenchyma suggestive of pulmonary contusions and/or hemorrhages were suspected. Mild pneumothorax and mild pleural effusion were also visible. A markedly displaced complete transverse diaphyseal fracture of the left humerus was also present.

**Figure 2:**
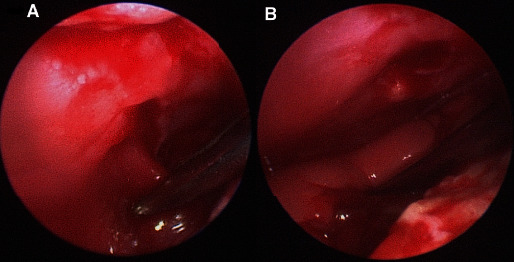
Thoracoscopic views with visualization of (A) the hernia and after the release of (B) the lung lobe.

Given the displacement observed at the level of the sternum and pain associated, surgical stabilization was elected. A ventral approach to the sternum was performed. After dissection of the subcutaneous tissue, the sternum was exposed. A tissue suspected to be of pulmonary origin was observed just to the right of the sternal dislocation due to tears at the level of pectoralis major muscle and intercostal muscles. Based on the concern for lung herniation, subxiphoid video-assisted thoracoscopy was performed. A Ternamian cannula (Karl Storz Ternamian EndoTip cannula) was positioned, and a rigid endoscope (5 mm, 30-degree oblique, 29 cm; Karl Storz) was placed. Small herniation (3 cm length) of a portion of the right caudal lung lobe (Figure 2(A)) was visualized at the level of muscle tears described above. The same subxiphoid incision was slightly extended, and a forceps was inserted without any instrument port. Using blunt dissection, the lung lobe was released under thoracoscopic guidance with concurrent distraction at the level of the dislocation (Figure 2(B)). The appearance of the lung was normal. No conventional leak test was performed. The 6^th^ sternebra was resected to facilitate the reduction of the dislocation. The dislocation was reduced using traumatic reduction forceps. The sternum was stabilized using an 8-hole 1.5 mm adaptation plate (VOI) with 6 cortical screws (all were 6 mm length). The length of the screws was determined on preoperative radiographs. The use of thoracoscopy in this case ensured no iatrogenic damage to the intrathoracic structures during sternal stabilization. A chest tube was then placed via a separate incision but closed to the xyphoid incision. The subxiphoid incision, pectoralis muscles, subcutaneous tissue, and skin were closed routinely. All the air present in the thorax was aspirated with the patient in dorsal recumbency, and the chest tube was left in place.

**Figure 3:**
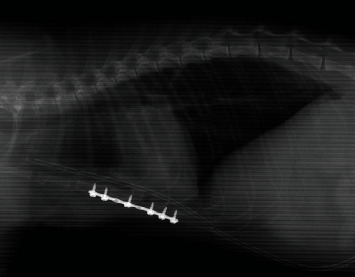
Right lateral postoperative thoracic radiographs showing proper positioning of the plate and screws. A chest tube is also present. Mild pleural effusion and small patchy areas of increased pulmonary opacity in the ventral and caudodorsal parts of the lung parenchyma remain.

Postoperative thoracic radiographs revealed good alignment of the sternum and proper positioning of the plate and screws (Figure 3).

The cat recovered uneventfully from anesthesia. The left humeral fracture was repaired 48 hours after the sternal stabilization. The chest tube was removed 24 hours after this second surgery.

The cat was discharged 2 days after chest tube removal. Meloxicam (Melosus Chat, Axience) was prescribed for 7 days (0.05 mg/kg per os q 24 hours) and amoxicillin-clavulanic acid (Synulox, Zoetis) for 7 days (20 mg/kg per os q 12 hr).

Two weeks following hospital discharge, the cat was evaluated. No postoperative complications were reported, and the physical examination was unremarkable at that time. Eight weeks postoperatively, the cat returned for radiologic assessment of the sternal and humeral repair. At that time, radiographic healing was deemed appropriate, and the cat's physical exam was unremarkable.

Final follow-up at 13 months postoperatively consisted of a phone consult with the owner. The cat was reportedly normal. Follow-up radiography was declined by the owner.

## 3. Discussion

There are no clear treatment guidelines concerning sternal stabilization in dogs and cats. In humans, a minority of sternal fracture/dislocation requires surgical management. Surgery is indicated in patients with significant displacement, uncontrolled pain, cardiorespiratory compromise, or clinical nonunion [10]. By extension in veterinary medicine, surgical management should be considered in cases of severe displacement, impaired breathing, or severe pain [3, 4]. Based on this case report, the authors also recommend surgical intervention if there is a clinical suspicion of incarcerated lung herniation.

Resection of the 6^th^ sternebra was preferred to complete reduction of the dislocation, as it was simpler to perform. In addition, our patient's thoracic cavity volume did not seem to be significantly affected by this reduction in the overall length of the sternum.

Various fixation techniques have been published in human medicine including stainless steel wires, plates, and internal cemented screws. The most appropriate technique involves using titanium plates and screws for plate osteosynthesis [11]. The adaptation plate has been chosen due to its size and because it was easily positioned on the sternum. We used cortical screws, but it is also possible to use locking screws. Locking plate technology could be beneficial in cancellous bone which is quite prominent in the sternebrae [4].

In all cats described in the literature, a traumatic origin is suspected as in the case of our patient. In the study of Van den Broek et al. [1], marked degenerative changes of the intersternebral joints were observed in 5 dogs just at the level of sternal subluxation. It might suggest spontaneous dislocation possible following subclinical instability and arthropathy of these joints.

Because of the risks associated with surgical repair of thoracic cavity, many veterinarians decide to manage these cases conservatively [5]. However, thoracoscopy is a valuable tool and could be used more systematically to limit intraoperative complications.

The authors were inspired by the study of Villagra et al. [12] in which video-assisted thoracoscopy was used to minimize the risk of intraoperative complication during pectus excavatum treatment in a dog. Thoracoscopy enabled us to diagnose and reduce the hernia. Moreover, it allowed us to ensure that no iatrogenic lung lesions were caused during screw placement.

Computed tomography seems indicated if concurrent injury to the thoracic organs is suspected [4]. In our case, it is not certain that the pulmonary hernia would have been clearly visualized on the CT scan because the hernia was very small. Focal discontinuity of the thoracic wall and irregularity of the ventral margins of the lung lobe would at least have been seen.

The most common lung hernia described in the literature is cervical lung hernia. Initially, cervical lung hernia was known as a rare phenomenon in dogs [13]. However, the study of Lee et al. [14] reported that cervical lung hernia is finally a common finding in older dogs. No surgical management has been performed in these cases. Indication for surgery is the presence of large hernias or incarcerated hernias [7, 15]. In our case, the lung hernia was incarcerated, as the hernia was reduced with concurrent distraction at the level of the dislocation. However, the hernia was very small, and it is difficult to state that the cat was clinical from this hernia rather than from its sternal dislocation. Another indication for surgery is the cause of the traumatism. Systematic bite wound explorative surgery at the level of thorax is recommended [9].

## 4. Conclusion

This case report illustrates the surgical repair of a sternal dislocation with a concomitant lung lobe hernia with the aid of video-assisted thoracoscopy in a cat. Surgical management of sternal dislocation should be considered in cases where severe displacement, impaired breathing, and severe pain are present. The authors recommend also surgical intervention if there is a suspicion of incarcerated lung herniation. Thoracoscopy could be included as part of the surgical management to reduce intraoperative risk.

## Data Availability

All data are available within the manuscript.
